# ROS-responsive biomaterial delivery of the TCM-derived small molecule coptisine protects stem cells and attenuates intervertebral disc degeneration

**DOI:** 10.3389/fbioe.2026.1945988

**Published:** 2026-09-22

**Authors:** Shouyun Xiao, Ninghang Ruan, Bijun Wang, Beiduo Shen, Ziqi Zhu, Feng Wang, Yufeng Huang

**Affiliations:** 1 Department of Spine Surgery, Shanghai East Hospital, School of Medicine, Tongji University, Shanghai, China; 2 Department of Orthopaedics, BenQ Medical Center, The Affiliated BenQ Hospital of Nanjing Medical University, Nanjing, Jiangsu Province, China; 3 Department of Orthopedics, Shanghai Sixth People’s Hospital, Shanghai Jiao Tong University School of Medicine, Shanghai, China

**Keywords:** coptisine, intervertebral disc degeneration, nucleus pulposus-derived mesenchymal stem cells, oxidative stress, ROS-responsive biomaterial

## Abstract

Oxidative stress compromise nucleus pulposus-derived mesenchymal stem cells (NPMSCs) and restrict endogenous repair in intervertebral disc degeneration (IDD). Here, human nucleus pulposus (NP) tissues showed that secreted phosphoprotein 1 (SPP1) expression increased with degeneration severity. Screening of 2,987 traditional Chinese medicine (TCM)-derived compounds, followed by cellular validation, identified coptisine as a candidate that reduced SPP1 expression while increasing NP-associated extracellular matrix components in NPMSCs. However, effective intradiscal delivery requires sustained local exposure within the adverse degenerative microenvironment. We therefore embedded coptisine-loaded poly (lactic-co-glycolic acid) microspheres in phenylboronic acid-modified gelatin methacryloyl to form an ROS-responsive depot (Cop@PLGA-GelMA-PBA). The composite showed sustained, oxidant-accelerated release and good cytocompatibility. Under H_2_O_2_ challenge, it preserved mitochondrial membrane potential and SOD2 abundance, while maintaining NP-associated extracellular matrix expression. Notably, SPP1 overexpression weakened these effects. When administered immediately after disc puncture, Cop@PLGA-GelMA-PBA maintained disc height and MRI signal, preserved tissue architecture and attenuated early degeneration. Collectively, these findings support responsive biomaterial delivery of a TCM-derived small molecule as a potential strategy for IDD.

## Introduction

Low back pain remains a leading cause of disability and rehabilitation need worldwide (Cieza et al., 2020). Intervertebral disc degeneration (IDD), a major structural contributor to this burden, involves progressive loss of nucleus pulposus (NP) hydration, extracellular matrix (ECM) imbalance, cellular dysfunction, and disruption of the annulus fibrosus and cartilaginous endplate (Risbud and Shapiro, 2014; Molinos et al., 2015). In the healthy NP, aggrecan (ACAN) and collagen II (Col-II) retain water and distribute compressive loads. As degeneration advances, matrix catabolism outpaces synthesis, disc height decreases, and intrinsic repair becomes inadequate (Guo et al., 2025). Current conservative and surgical treatments can relieve symptoms or stabilize the motion segment but do not restore disc biology (Risbud and Shapiro, 2014). Strategies that preserve resident reparative cells and rebuild the NP matrix are therefore needed (Liu et al., 2020).

Inflammation and oxidative stress (OS) reinforce one another within the degenerating disc (Kang et al., 2022; Wang et al., 2025). Excess reactive oxygen species (ROS) disrupt mitochondrial homeostasis, accelerate senescence and cell death, and promote matrix-catabolic programs (Feng et al., 2017; Wang F. et al., 2023). TNF-α and IL-1β activate NF-κB, MAPK, inflammasome-associated, and related pathways, suppressing matrix synthesis while amplifying ECM degradation (Molinos et al., 2015; Chen et al., 2022). Degeneration also depletes or impairs resident progenitor and mesenchymal populations (Liu et al., 2017; Sakai et al., 2012). Acidity and restricted nutrient transport further constrain their reparative function (Liu et al., 2017; Urban et al., 2004). Moreover, limited transport within the largely avascular disc restricts systemic drug exposure. Local delivery systems that respond to pathological cues may therefore be particularly suitable for IDD.

Secreted phosphoprotein 1 (SPP1), which encodes osteopontin, participates in cell adhesion, matrix remodeling, immune-cell recruitment, and mitochondrial stress responses (Yim et al., 2022). Its function in the disc appears to depend on developmental stage, cell state, and tissue compartment. During early disc formation, notochord-derived SPP1 supported chondrogenic programs and restricted vascular cues, whereas a recent study of adult degenerative NP linked increased SPP1 to ITGα5/β1-dependent mitophagy impairment, calcification, and matrix loss (Gu et al., 2025; Zhou et al., 2023). Osteopontin deficiency can also aggravate cartilaginous endplate degeneration (Wang et al., 2024). These observations argue against treating SPP1 as uniformly protective or deleterious throughout the disc. We therefore used its increase in adult degenerative NP as a disease-associated signal for compound selection and tested the resulting response directly in NPMSCs.

TCM-derived small molecules offer broad chemical diversity and often act across interconnected inflammatory, redox, senescence, and cell-death pathways (Wu et al., 2025; Shi et al., 2026). In IDD models, Quercetin has modulated Nrf2/NF-κB-dependent senescence, Panax notoginseng saponins have protected NP cells from oxidative injury, and Isoginkgetin delivery has coupled ROS control to autophagy (Guo et al., 2024; Yu et al., 2023). These studies support natural products as a source of multi-pathway candidates, but they do not establish interchangeability among compounds. Coptisine is a protoberberine alkaloid found in Coptis chinensis (Lu et al., 2023), studies outside the disc have reported anti-inflammatory, mitochondrial, and redox-modulatory effects (Friedemann et al., 2015; Hu et al., 2019). However, whether coptisine counteracts oxidative injury to regulate SPP1 expression and preserve NP-associated ECM in NPMSCs—and thus delays the progression of IDD—remains to be established.

Its activity in NPMSCs and IDD has not been established. Systemic use may be limited by unfavorable exposure and a concentration-dependent safety window (Lu et al., 2023). Independently, restricted transport and loss of locally administered payloads from the disc favor a retention-oriented delivery strategy (Wang et al., 2026). We therefore combined disease-informed compound selection with a local carrier designed to retain coptisine and release it preferentially in an oxidizing environment. Stimuli-responsive biomaterials can couple drug release to local pathology rather than to time alone (Guo et al., 2025; Zhang et al., 2026). PLGA microspheres provide sustained payload delivery (Xiong et al., 2025), whereas GelMA forms a photocrosslinkable, cell-compatible porous matrix (Yang et al., 2025). Phenylboronic-acid-containing networks have been used for microenvironment-responsive intradiscal delivery (Wang et al., 2026; Lan et al., 2025; Gao et al., 2024), while broader studies define the ROS-responsive chemistry of PBA polymers (Zeng et al., 2021). ROS-responsive microspheres and hydrogels have reduced oxidative injury in experimental IDD (Xu et al., 2026). This chemistry is relevant to IDD, where OS is both a pathogenic factor and a potential release trigger. However, a GelMA-PBA-PLGA carrying coptisine has not been evaluated in NPMSCs or IDD.

In this study, human specimens were used to identify and validate SPP1 as a degeneration-associated discovery signal. We screened 2,987 TCM-derived compounds, prioritized coptisine by computational and cellular testing, and incorporated coptisine-loaded PLGA microspheres into GelMA-PBA (Cop@PLGA-GelMA-PBA). We then examined material microstructure, ROS-responsive release and degradation, cytocompatibility, mitochondrial and matrix-associated responses in NPMSCs, the effect of SPP1 overexpression, and treatment-associated transcriptomic changes. The formulation was finally assessed as an immediate post-injury intervention in a puncture-induced rat model of IDD. The advance lies in integrating an adult degeneration-associated signal with TCM-derived small-molecule selection and ROS-responsive local delivery.

## Experimental section

### Human intervertebral disc specimens

NP specimens were collected at Shanghai East Hospital from patients treated surgically for lumbar disc herniation with spinal canal stenosis. Every participant gave written informed consent, and the hospital ethics committee authorized the protocol. Qualified individuals were 18–70 years old, and tissue was sampled at L3/4, L4/5 or L5/S1. Two radiologists who had no access to the tissue findings independently assigned Pfirrmann grades from preoperative T2-weighted MRI (Pfirrmann et al., 2001). An agreed grade was used directly; discordant readings were averaged for grouping. Specimens were used for histology and SPP1 immunofluorescence and RT–qPCR.

### Histology and SPP1 immunofluorescence of human tissues

Immediately after collection, NP tissue was photographed and immersed in 4% paraformaldehyde for 24 h. Paraffin blocks were prepared and sectioned at 4 µm. Tissue architecture, fibrosis, cell abundance, and proteoglycan-rich matrix were examined by H&E and Safranin O staining. For SPP1 immunofluorescence, antigen retrieval was followed by blocking, incubation with anti-SPP1 antibody (1:200; Abcam, Cambridge, United Kingdom), fluorophore-labeled secondary antibody, and DAPI. Acquisition parameters were held constant across specimens.

### Traditional Chinese medicine monomer library screening and molecular docking

The Traditional Chinese Medicine Active Compound Library (HY-L065; MedChemExpress, NJ, United States), containing 2,987 compounds, was screened against a three-dimensional structural model assigned to rat SPP1/osteopontin. P08721 (OSTP_RAT) is the UniProt sequence accession used to identify the rat protein. Protein and ligand structures were prepared using a standard structure-based virtual-screening workflow. Compounds were ranked by predicted docking affinity, with more negative values indicating a more favorable predicted interaction. The ten highest-ranked candidates were taken forward for cell testing.

### NPMSC isolation and culture

Sprague–Dawley (SD) rats were euthanized by an intraperitoneal overdose of sodium pentobarbital (150 mg kg^−1^). Death was confirmed by the absence of spontaneous respiration, heartbeat, and corneal reflex before tissue collection. NP tissue from SD rats (6–8 weeks old) was used to obtain NPMSCs. Fragmented NP tissue underwent digestion in 0.2% collagenase type II (Gibco) for 6 h at 37 °C. After centrifugation at 800 *g* for 5 min, cells and partial tissue debris were placed in Cyagen mesenchymal stem-cell medium containing 20% fetal bovine serum (HyClone) and 1% penicillin–streptomycin. Cultures were maintained at 37 °C (2% O_2_, 5% CO_2_, balance N_2_). Medium replacement on the next day removed non-adherent cells and debris; changes were made twice a week. At 80%–90% confluence, cells were subcultured 1:3. Passage-3 cells from independent preparations were used throughout the characterization and studies.

### NPMSC characterization

Passage-3 cultures were inspected by microscopy. Mesenchymal stromal-cell properties were evaluated with reference to ISCT criteria (Dominici et al., 2006). Osteogenic, adipogenic, and chondrogenic induction was performed with lineage-specific Cyagen kits. Cells were plated in six-well dishes at 5 × 10^^5^ cells mL^−1^, and induction began when cultures reached approximately 80% confluence. At the designated endpoint, cells were fixed in 4% paraformaldehyde. Alizarin Red S, Oil Red O, and Alcian Blue were used to visualize osteogenic, adipogenic, and chondrogenic differentiation, respectively. CD34, CD45, CD73, CD90, and CD105 were examined by immunofluorescence. Primary antibodies were applied for 2 h at room temperature (1:500; Abcam; available catalogue numbers ab150077, ab150078, ab150080, and ab175471), followed by fluorescent secondary antibodies and DAPI. Cells were observed and imaged using a fluorescence microscope.

### Candidate-compound testing

For experimental prioritization, NPMSCs were incubated for 24 h with each of the ten leading docking candidates at 1, 10, and 100 μM. SPP1 transcript abundance was then compared by RT–qPCR. Coptisine chloride (MedChemExpress, HY-N0736) yielded the preferred expression profile and was advanced to validation. A subsequent experiment exposed NPMSCs to 100 µM coptisine chloride for 24 h and measured SPP1, COL-II, and ACAN mRNA. Immunofluorescence was used to assess SPP1 and ACAN protein. Controls received the corresponding vehicle. Three independent cell preparations were included in every experiment.

### RNA extraction and RT–qPCR

RNA was purified with a column-based kit (Beyotime, Shanghai, China), after which concentration and purity were determined spectrophotometrically. Reverse transcription used the Quantscript RT Kit (Applied Biological Materials, Richmond, BC, Canada). Reactions for quantitative PCR contained ChamQ Universal SYBR qPCR Master Mix (Vazyme, Nanjing, China) and were run on a Bio-Rad instrument. Fold changes were obtained with the 2^−ΔΔCT^ calculation (Livak and Schmittgen, 2001).

### Preparation of coptisine-loaded PLGA microspheres, GelMA-PBA and composite formation

Coptisine-loaded PLGA (Cop@PLGA) microspheres were produced by adapting our previously reported oil-in-water emulsion and solvent-evaporation procedure (Wang F. et al., 2023). Ester-terminated 50:50 PLGA (30–60 kDa; 100 mg) was dissolved in 4.5 mL dichloromethane. Separately, 10 mg coptisine chloride was prepared in 0.5 mL dimethyl sulfoxide and combined with the PLGA solution. This organic phase was introduced dropwise into 100 mL of 1% poly (vinyl alcohol) while homogenizing at 10,000 rpm for 2 min. Solvent removal proceeded during 18 h of stirring. Particles were recovered at 4 °C by centrifugation (12,000 rpm, 20 min), rinsed three times with deionized water, and freeze-dried. GelMA-PBA (Engineering for Life, Suzhou, China) was used as the hydrogel component. Previous work has established PBA-modified GelMA as a basis for pathological-microenvironment-responsive IDD formulations [46]. For the present formulation, GelMA-PBA was prepared at 10% (w/v) in PBS and mixed with lithium phenyl-2,4,6-trimethylbenzoylphosphinate at 5 mg mL^−1^ (0.5% w/v). Cop@PLGA was added at 5 mg mL−1 (0.5% w/v). Exposure to 405 nm light for 30 s generated Cop@PLGA-GelMA-PBA. The unloaded comparator used blank PLGA microspheres in the otherwise identical GelMA-PBA precursor.

### Material characterization

Lyophilized PLGA and hydrogels were fractured under liquid nitrogen, coated with gold, and examined using a HITACHI S-4800 scanning electron microscope. ImageJ was used to determine particle diameter and pore size. Reverse-phase HPLC-DAD was used to monitor coptisine at 345 nm. Chromatography used a 4.6 × 250 mm C18 column (5 µm), an acetonitrile/0.1% formic-acid aqueous phase (30:70, v/v), a 1.0 mL min−1 flow rate, and a column temperature of 30 °C. Release and mass-loss studies were conducted at 37 °C in PBS or PBS containing 100 µM tert-butyl hydroperoxide (TBHP). At scheduled times over 21 days, release medium was sampled and replenished. Cumulative release was normalized to encapsulated coptisine. For degradation measurements, initial and post-incubation lyophilized masses were used to calculate the fraction remaining.

### Cytocompatibility

Cytocompatibility was evaluated using material-conditioned media. NPMSCs received standard complete medium, extraction medium from GelMA-PBA, or from Cop@PLGA-GelMA-PBA. Each extract was generated by incubating the relevant material in complete medium for 48 h. Live/Dead staining (Abcam) was carried out on culture days 3 and 7. Cell viability was expressed as the fraction of all cells that were live. Proliferative activity was assessed at the same time points with CCK-8, and absorbance was read at 450 nm. Three independent material batches were used.

### Oxidative challenge and SPP1 overexpression

The oxidative-challenge experiment comprised untreated cells (Control group), H_2_O_2_ alone (H_2_O_2_ group), H_2_O_2_ followed by Cop@PLGA-GelMA-PBA (H_2_O_2_+ Cop@PLGA-GelMA-PBA group), and the same material intervention after SPP1 overexpression (H_2_O_2_+SPP1-OE + Cop@PLGA-GelMA-PBA group). GeneChem (Shanghai, China) supplied LV-SPP1 and its negative-control construct (LV-NC). According to the manufacturer’s instructions and preliminary optimization experiments, passage-3 NPMSCs were transduced with lentiviruses encoding rat SPP1 (LV-SPP1) or a matched negative-control vector (LV-NC) at a multiplicity of infection (MOI) of 20. NPMSCs were seeded in six-well plates and transduced at approximately 40%–50% confluence. After 12 h of incubation, the viral medium was replaced with fresh complete medium. Cells were cultured for a further 72 h before subsequent experiments. SPP1 immunofluorescence staining was performed to verify successful SPP1 overexpression. Cells then received 100 µM H_2_O_2_ for 12 h and Cop@PLGA-GelMA-PBA for 24 h at a coptisine-equivalent concentration of 100 µM.

### JC-1 and immunofluorescence assays

JC-1 staining was used to estimate mitochondrial membrane potential according to the supplier’s protocol (Smiley et al., 1991). Aggregate-associated red and monomer-associated green fluorescence were recorded under unchanged imaging settings. After background correction, the red-to-green fluorescence ratio was calculated. Separate immunofluorescence assays detected SOD2, Col-II, and ACAN with antibodies specified. Mean fluorescence was determined in ImageJ from at least three random fields per sample. JC-1 was treated as an indicator of mitochondrial polarization, and SOD2 staining as a measure of antioxidant-enzyme abundance.

### RNA sequencing and enrichment analysis

Transcriptome profiling used RNA from untreated NPMSCs and NPMSCs incubated for 24 h with Cop@PLGA-GelMA-PBA. Each condition comprised three independent cell preparations. Poly(A)-selected libraries were sequenced as 150-bp paired-end reads on an Illumina NovaSeq 6,000, with an intended depth of approximately 40 million clean read pairs per sample. Fastp v0.23.2 was used for read filtering; HISAT2 v2.2.1 aligned reads to Rnor_6.0; and featureCounts v2.0.3 produced gene-level counts. DESeq2 v1.36.0 identified differential expression (Shi et al., 2026). The prespecified cutoff was |log2 fold change| > 1 with Benjamini–Hochberg-adjusted *P* < 0.05. GO and KEGG over-representation analyses were run in cluster Profiler v4.4.4 against the background of all expressed genes, using adjusted *P* < 0.05 (Guo et al., 2024).

### Puncture-induced IDD model and material injection

The Institutional Animal Care and Use Committee of Shanghai East Hospital approved the animal protocol and reporting follows ARRIVE guidance (Percie du Sert et al., 2020). SD rats (3 months old; 250–300 g; both sexes) were randomly assigned to an uninjured control (Control group, n = 5), puncture only (Puncture group, n = 5), injection of unloaded PLGA-GelMA-PBA following puncture (P + PLGA-GelMA-PBA group, n = 5), or injection of Coptisine@PLGA-GelMA-PBA following puncture (P + Cop@PLGA-GelMA-PBA group, n = 5). Rats were anesthetized intraperitoneally with 1% pentobarbital sodium at 0.4 mL kg^−1^. Following radiographic localization of Co6/7, a 21-G needle was advanced percutaneously to 5 mm in accordance with a rat-tail puncture approach (Han et al., 2008). The assigned 2 µL injection was delivered immediately through a 31-G needle, which remained in place for 5 min to reduce reflux. Analgesia was provided for 3 days after surgery. Because dosing occurred directly after injury, the model addressed early intervention rather than established degeneration.

### Radiography and MRI

Standardized lateral radiographs were obtained at weeks 4 and 8 with an MCR-6000 molybdenum-target system. Image acquisition and quantification followed our earlier protocol (Wang F. et al., 2023). Anterior, central, and posterior disc heights were averaged and divided by the average height of the two neighboring vertebral bodies to obtain disc height index (DHI). Values were normalized to each animal’s baseline and reported as DHI%. T2-weighted MRI used a 3.0-T GE Healthcare scanner (repetition time 3,000 m; echo time 60 m; nine excitations; 1.5-mm slices; 5-cm field of view). The MRI index, also calculated as previously described (Wang F. et al., 2023), was the product of NP cross-sectional area and mean T2 signal intensity. Identical regions of interest were analyzed by two observers who were unaware of treatment allocation.

### Animal histology and immunofluorescence

SD rats were euthanized by an intraperitoneal overdose of sodium pentobarbital (150 mg kg^−1^). Death was confirmed by the absence of spontaneous respiration, heartbeat, and corneal reflex before tissue collection. Discs collected at week 8 were fixed, decalcified, dehydrated, embedded in paraffin, and cut sagittally at 4 µm. H&E and Safranin O/Fast Green staining were used for morphology. Two observers blinded to allocation assigned degeneration grades using the rat-tail puncture framework of Han et al. (2008). For fluorescence analysis, sections were deparaffinized, subjected to antigen retrieval and blocking, and incubated with rabbit antibodies to ACAN or Col-II (1:150; Proteintech, Wuhan, China). Fluorophore-linked secondary antibodies and DAPI were then applied. Sections were observed and imaged using a fluorescence microscope. ImageJ-based quantification used background subtraction and a consistent threshold to calculate relative fluorescence.

### Statistical analysis

The data were summarized as mean ± standard error of the mean (SEM). Statistical analyses were performed using SPSS 19.0 (SPSS Inc., Chicago, IL, United States). Data normality was assessed via Shapiro-Wilk test prior to selecting parametric methods. Depending on data distribution and group comparisons, intergroup differences were evaluated using Student’s t-test or one-way ANOVA with Tukey’s honest significant difference (HSD) *post hoc* test. For non-normally distributed data, the Mann–Whitney U or Kruskal–Wallis H test was employed as appropriate. Statistical significance was set at *P* < 0.05. Figure symbols represent *P* < 0.05 (*), *P* < 0.01 (**), *P* < 0.001 (***), and *P* < 0.0001 (****).

## Results

### SPP1 expression is associated with human disc degeneration

Representative T1-and T2-weighted MRI and gross NP specimens illustrated the contrast between Pfirrmann Grade II and Grade IV degeneration (Figure 1A). Compared with Grade II tissue, Grade IV tissue showed pronounced structural disorganization, fibrosis, reduced cellularity, and loss of Safranin O staining (Figure 1B). SPP1 immunofluorescence was visibly stronger in Grade IV specimens (Figure 1C). SPP1 mRNA was significantly higher in Grade IV–V than in Grade II–III NP tissue (*P* < 0.05, Figure 1D).

**FIGURE 1:**
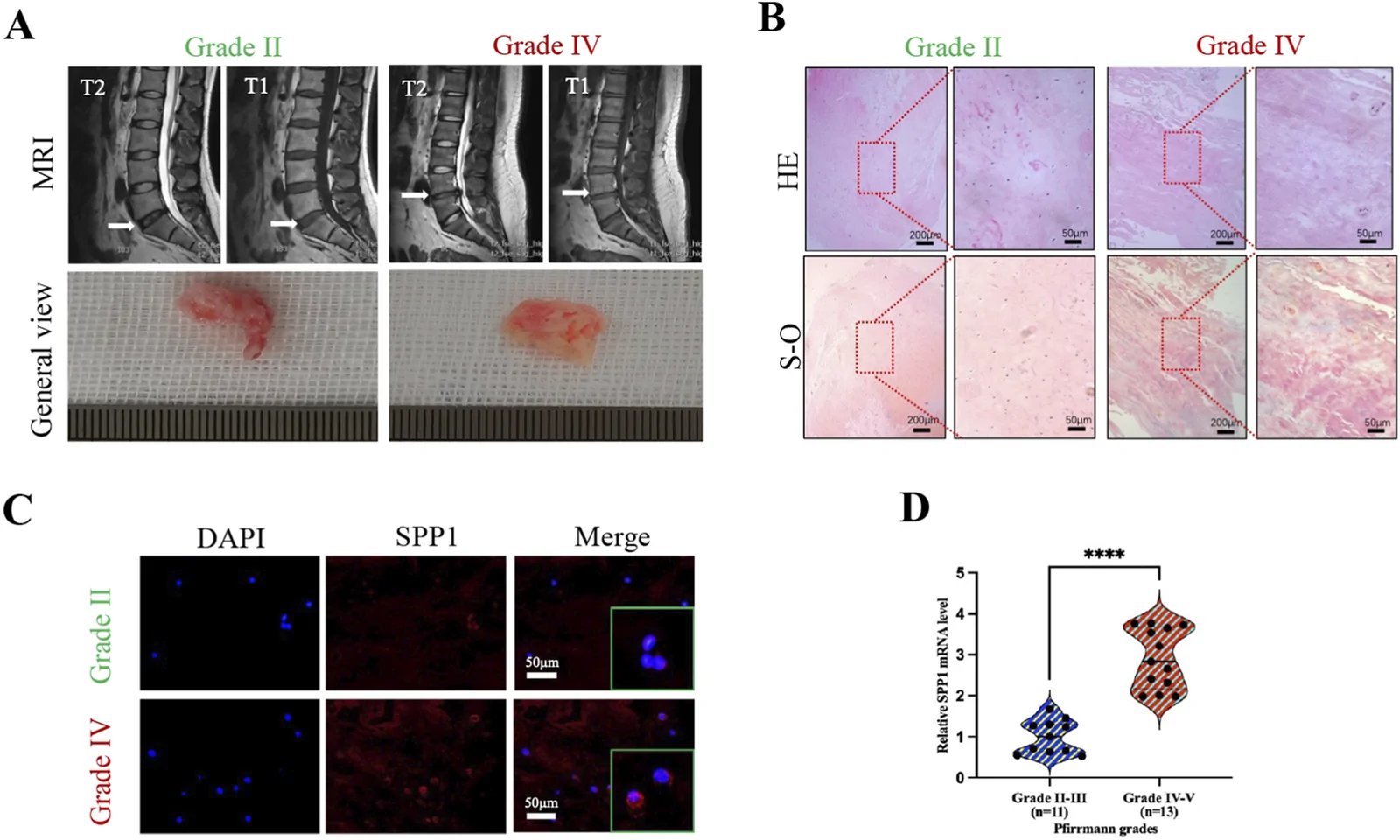
SPP1 expression increases with the severity of human intervertebral disc degeneration. **(A)** Representative T2-and T1-weighted lumbar MRI from patients with Pfirrmann Grade II or Grade IV degeneration (upper panels) and the corresponding gross NP specimens (lower panels). White arrows indicate the sampled discs. **(B)** Representative H&E and Safranin O staining showing greater fibrosis, lower cellularity, and matrix loss in Grade IV tissue. **(C)** Representative SPP1 immunofluorescence showing stronger SPP1 staining (red) in Grade IV than in Grade II tissue; nuclei were counterstained with DAPI (blue). Scale bar, 50 µm. **(D)** SPP1 mRNA measured by RT–qPCR in an independent cohort (Grades II–III, n = 11; Grades IV–V, n = 13). Data are mean ± SEM; unpaired two-tailed Student’s t-test. All data are expressed as the mean ± SEM, *, *p* < 0.05; **, *p* < 0.01; ***, *p* < 0.001, ****, *p* < 0.0001.

### Integrated computational and experimental screening prioritizes coptisine

The screening strategy combined virtual docking, candidate ranking, and molecular validation (Figure 2A). Docking of 2,987 TCM-derived compounds against a structural model assigned to the rat SPP1 sequence yielded a broad range of predicted affinities (Figure 2B); the ten most favorable candidates (Benzo [a]pyrene, Roemerine, 6-Methoxydihydrosanguinarine, Triptonoterpene, Dehydro-α-lapachone, Zearalenone, Coptisine, Graveoline, Ellipticine (hydrochloride), Ellipticine) were selected for cell testing (Figure 2C). Their effects on SPP1 expression differed across compounds and concentrations, with coptisine producing the most consistent reduction (Figure 2D). At the selected concentration (100 µM), coptisine decreased SPP1 mRNA and increased COL2A1 and ACAN mRNA relative to vehicle (Figure 2E). The computationally predicted coptisine–model pose is shown in Figure 2F. Immunofluorescence showed lower SPP1 and higher ACAN protein signals after coptisine treatment (Figures 2G,H). Computational prioritization and cellular testing therefore converged on coptisine.

**FIGURE 2:**
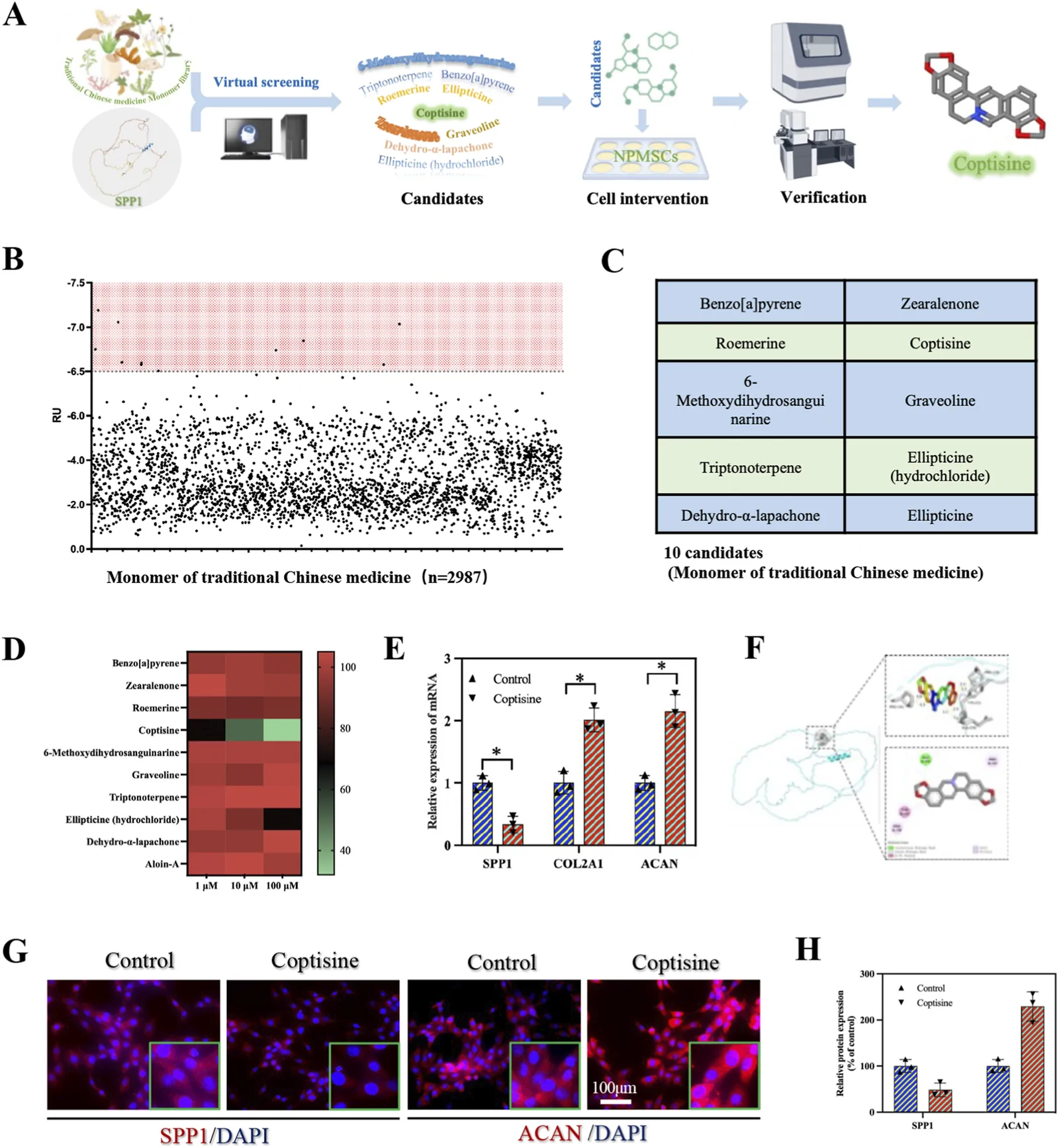
Computational screening and cellular validation identify coptisine as an SPP1-associated TCM-derived small molecule. **(A)** Workflow for virtual screening, candidate ranking, NPMSC treatment, and validation. **(B)** Distribution of predicted docking affinities (kcal mol−1) for 2,987 TCM-derived compounds against a structural model assigned to the rat SPP1 sequence; more negative values indicate more favorable predicted interactions. **(C)** Ten highest-ranked compounds. **(D)** Heat map of SPP1 mRNA after treatment with each candidate at 1, 10, or 100 µM for 24 h **(E)** SPP1, COL2A1, and ACAN mRNA in vehicle- and coptisine-treated NPMSCs. **(F)** Predicted pose of coptisine in the structural model. **(G)** Representative SPP1 and ACAN protein immunofluorescence after coptisine treatment. **(H)** Quantification of the fluorescence signals in **(G)** n = 3 independent cell preparations; data are mean ± SEM. Statistical symbols are defined in the figure. Docking was used for prioritization and does not establish direct binding. *, *p* < 0.05; **, *p* < 0.01; ***, *p* < 0.001, ****, *p* < 0.0001.

### Cop@PLGA-GelMA-PBA provides ROS-responsive release and good cytocompatibility

SEM showed spherical Cop@PLGA microspheres, an interconnected porous GelMA-PBA network, and microspheres distributed throughout the composite, the Cop@PLGA-GelMA-PBA group revealed a homogeneous distribution of drug-loaded microspheres within the GelMA-PBA network (Figure 3A). Coptisine was released progressively over 21 days, with faster release in TBHP-containing medium than in PBS (Figure 3B). Material mass also declined more rapidly in TBHP, indicating oxidation-accelerated degradation under the tested *in vitro* conditions (Figure 3C). Microsphere diameters were approximately normally distributed around 30–35 µm (Figure 3D), and GelMA-PBA and Cop@PLGA-GelMA-PBA had similar pore-size distributions centered at approximately 85–95 µm (Figure 3E). Live/dead staining showed predominantly viable NPMSCs in all three conditions on days 3 and 7 (Figure 3F), with no significant between-group difference in viability (Figure 3G). CCK-8 absorbance increased from day 3 to day 7 in every group, with no evident suppression by either formulation (Figure 3H). These assays establish oxidation-sensitive behavior and short-term cytocompatibility *in vitro*.

**FIGURE 3:**
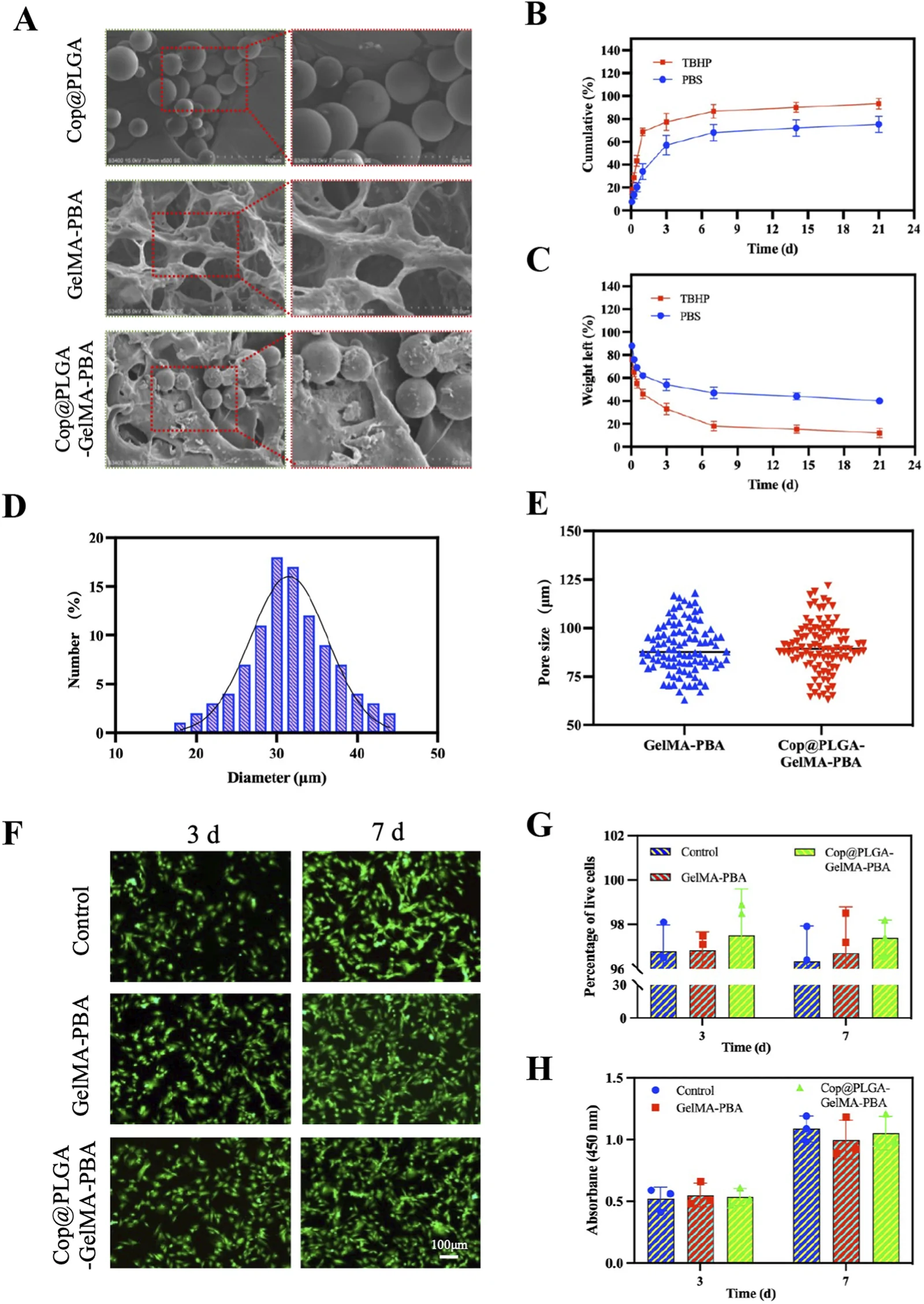
Microstructure, ROS-responsive behavior, and cytocompatibility of Cop@PLGA-GelMA-PBA. **(A)** SEM images of Cop@PLGA microspheres, GelMA-PBA, and Cop@PLGA-GelMA-PBA. **(B)** Cumulative coptisine release from Cop@PLGA-GelMA-PBA in PBS or 100 µM tert-butyl hydroperoxide (TBHP). **(C)** Residual material mass under the same conditions. **(D)** Cop@PLGA particle-size distribution. **(E)** Pore-size distributions of GelMA-PBA and Cop@PLGA-GelMA-PBA. **(F)** Live/dead staining of NPMSCs in control medium, GelMA-PBA extract, or Cop@PLGA-GelMA-PBA extract at days 3 and 7. Scale bar, 100 µm. **(G)** Quantification of live-cell percentage; no significant differences were detected among groups. **(H)** CCK-8 absorbance at days 3 and 7.

### Isolated cells exhibit an NPMSC-like phenotype

Passage-3 cells adhered to plastic and displayed the spindle-shaped morphology typical of mesenchymal stromal cells (Figure 4A). Lineage induction produced Alizarin Red positive mineral deposits, Oil Red O-positive lipid droplets, and Alcian Blue-positive glycosaminoglycan-rich matrix (Figure 4B). Immunofluorescence showed weak CD34 and CD45 staining and strong CD73, CD90, and CD105 staining (Figure 4C). Taken together, morphology, trilineage differentiation, and the qualitative marker pattern supported an NPMSC-like identity.

**FIGURE 4:**
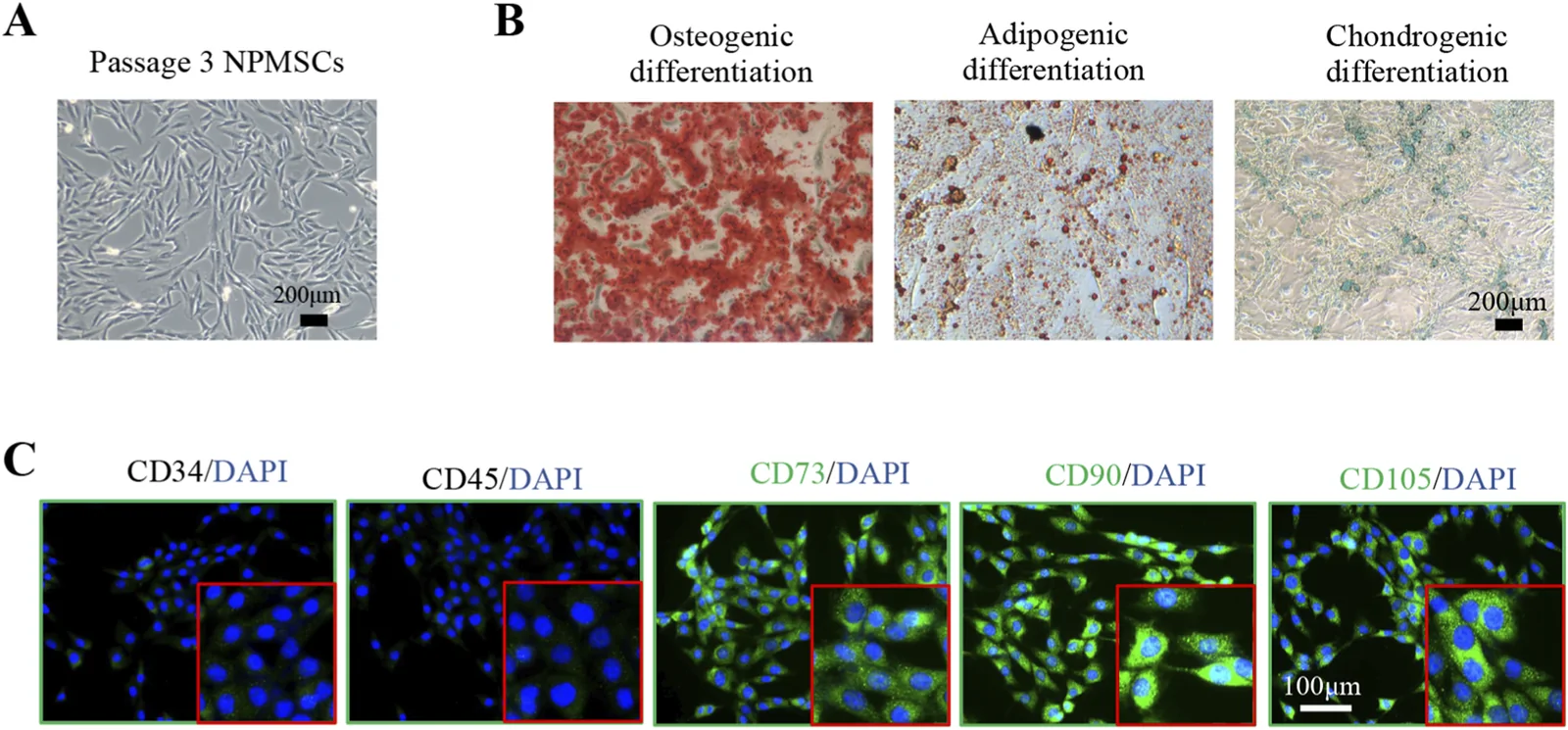
Isolation and characterization of rat NPMSCs. **(A)** Representative passage-3 NPMSCs showing spindle-shaped morphology. **(B)** Osteogenic, adipogenic, and chondrogenic differentiation demonstrated by Alizarin Red S, Oil Red O, and Alcian Blue staining, respectively. **(C)** Immunofluorescence showing weak CD34 and CD45 signals and strong CD73, CD90, and CD105 signals; nuclei were counterstained with DAPI.

### The loaded composite preserves mitochondrial and matrix-associated phenotypes during oxidative challenge

H_2_O_2_ reduced the JC-1 aggregate-to-monomer ratio, consistent with mitochondrial depolarization, Cop@PLGA-GelMA-PBA partially restored the ratio, whereas SPP1 overexpression blunted this effect (Figures 5A,B). H_2_O_2_ also lowered SOD2 fluorescence, which recovered after treatment with the loaded composite but declined again when SPP1 was overexpressed (Figures 5C,D). Col-II and ACAN showed the same response pattern (Figures 5E–G). Within the four-group design, these findings indicate that the loaded composite preserved mitochondrial polarization, antioxidant-enzyme abundance, and matrix-associated proteins during oxidative challenge, and that SPP1 overexpression opposed these responses. To verify lentiviral transduction and SPP1 overexpression, rat NPMSCs were transduced with LV-SPP1 or LV-NC. RFP fluorescence was observed in both groups, supporting successful lentiviral transduction under the selected conditions (Figure 5H). In addition, SPP1 immunofluorescence was visibly stronger in the LV-SPP1 group than in the LV-NC group, indicating increased SPP1 protein expression after LV-SPP1 transduction (Figure 5I).

**FIGURE 5:**
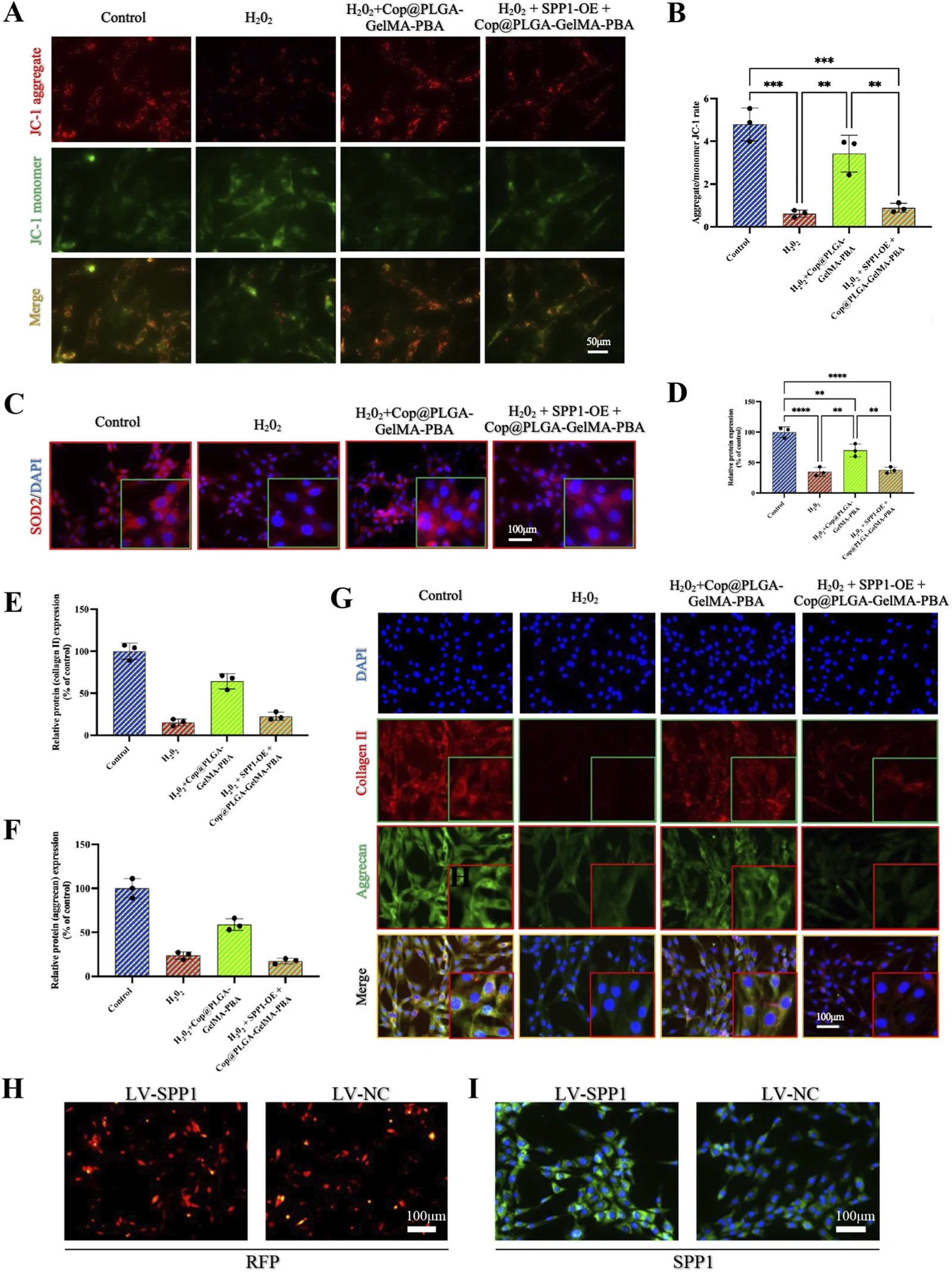
Cop@PLGA-GelMA-PBA preserves mitochondrial and matrix-associated phenotypes through an SPP1-associated response. **(A)** JC-1 aggregate (red), monomer (green), and merged fluorescence. **(B)** JC-1 aggregate-to-monomer ratio. **(C)** Representative SOD2 immunofluorescence. **(D)** SOD2 fluorescence intensity. **(E,F)** Col-II and ACAN fluorescence intensity. **(G)** Representative Col-II (red) and ACAN (green) immunofluorescence. Nuclei were counterstained with DAPI. **(H)** Representative images show RFP reporter fluorescence in NPMSCs transduced with LV-SPP1 or the matched negative-control vector (LV-NC). **(I)** Immunofluorescence staining showed increased SPP1 expression in LV-SPP1-transduced NPMSCs compared with LV-NC-transduced cells. All data are expressed as the mean ± SEM, *, *p* < 0.05; **, *p* < 0.01; ***, *p* < 0.001, ****, *p* < 0.0001.

### Transcriptomic profiling identifies inflammation- and stress-related enrichment signatures

RNA sequencing compared untreated and Cop@PLGA-GelMA-PBA-treated NPMSCs. Treatment was associated with broad transcriptional differences, with SPP1 among the differentially expressed genes (Figure 6A). Gene Ontology analysis identified enrichment of terms related to nutrient responses, interleukin-1, glucocorticoids, bacterial-origin molecules, lipopolysaccharide, biotic stimuli, and leukocyte migration (Figure 6B). KEGG analysis similarly identified enrichment of cytokine–cytokine receptor interaction, TNF, chemokine, MAPK, IL-17, NF-κB, AGE–RAGE, integrin, cell-adhesion, and ferroptosis-related pathways (Figure 6C). These enrichment signatures indicate that the transcriptional response to the composite was associated with inflammation- and stress-related biological programs; they do not establish the direction of pathway regulation or a specific anti-inflammatory effect.

**FIGURE 6:**
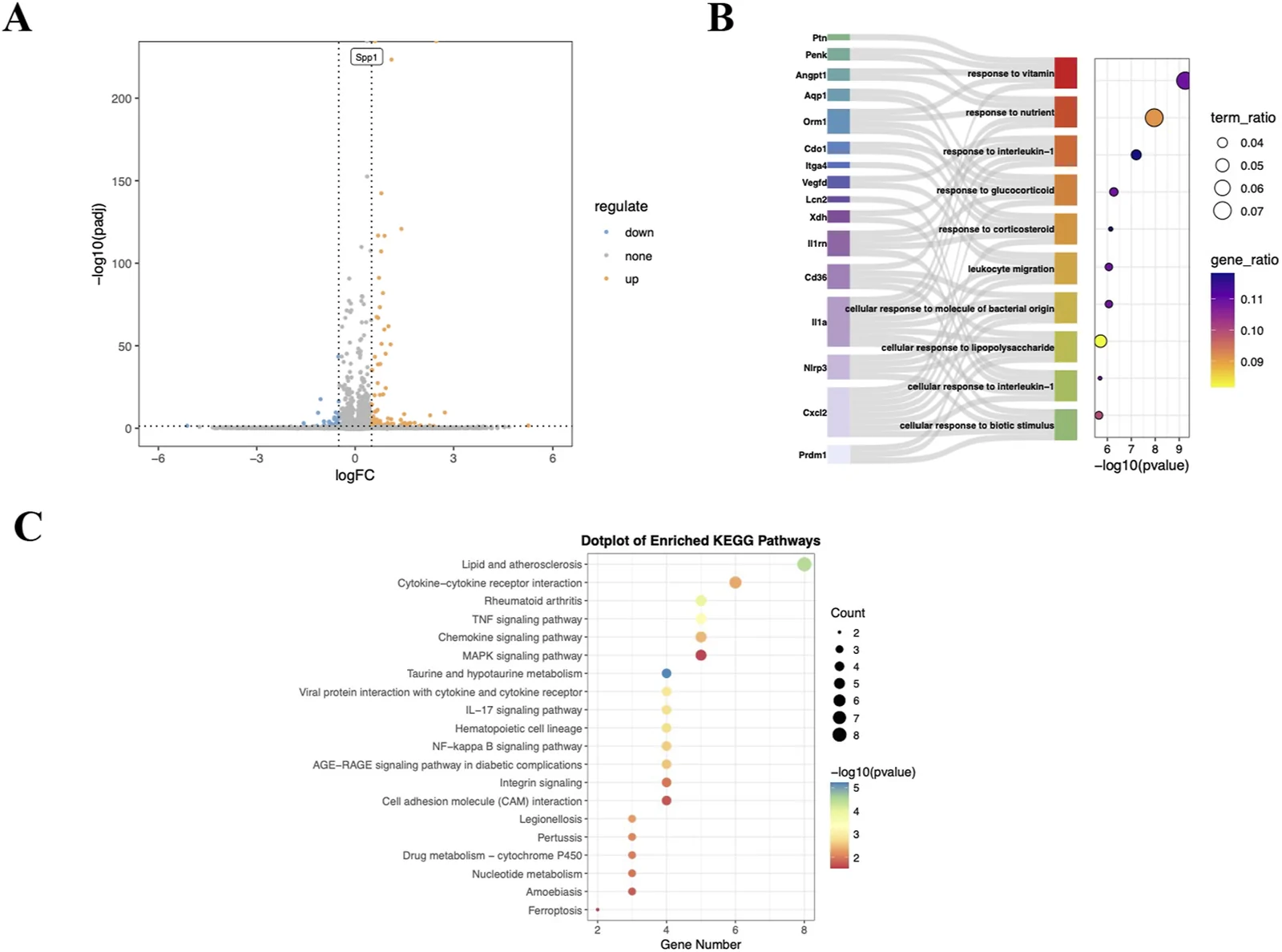
Transcriptomic response of NPMSCs to Cop@PLGA-GelMA-PBA. Control and loaded-composite groups were compared using three independent biological replicates per group. **(A)** Volcano plot of differentially expressed genes; SPP1 is highlighted. Dotted lines indicate the fold-change and significance thresholds. **(B)** GO gene–term association diagram and enrichment dot plot. Dot size denotes the term ratio, dot color denotes the gene ratio, and the horizontal axis shows −log10(P value). **(C)** KEGG enrichment dot plot. Dot size denotes gene count, dot color denotes −log10(P value), and the horizontal axis shows gene number.

### Cop@PLGA-GelMA-PBA preserves disc height and MRI signal after puncture

Radiography and MRI were performed 4 and 8 weeks after puncture and immediate injection (Figure 7A). Disc height decreased in every injured group relative to the control; however, the reduction was significantly attenuated in the P + Cop@PLGA-GelMA-PBA group compared with both the puncture-only and P + PLGA-GelMA-PBA groups at both time points (Figures 7B,D,E). T2-weighted NP signal was likewise markedly reduced by puncture. MRI indices remained higher in the P + Cop@PLGA-GelMA-PBA group than in the Puncture and P + PLGA-GelMA-PBA groups at weeks 4 and 8 (Figures 7C,D,F). Thus, coptisine loading improved imaging outcomes relative to the carrier alone when delivered immediately after injury.

**FIGURE 7:**
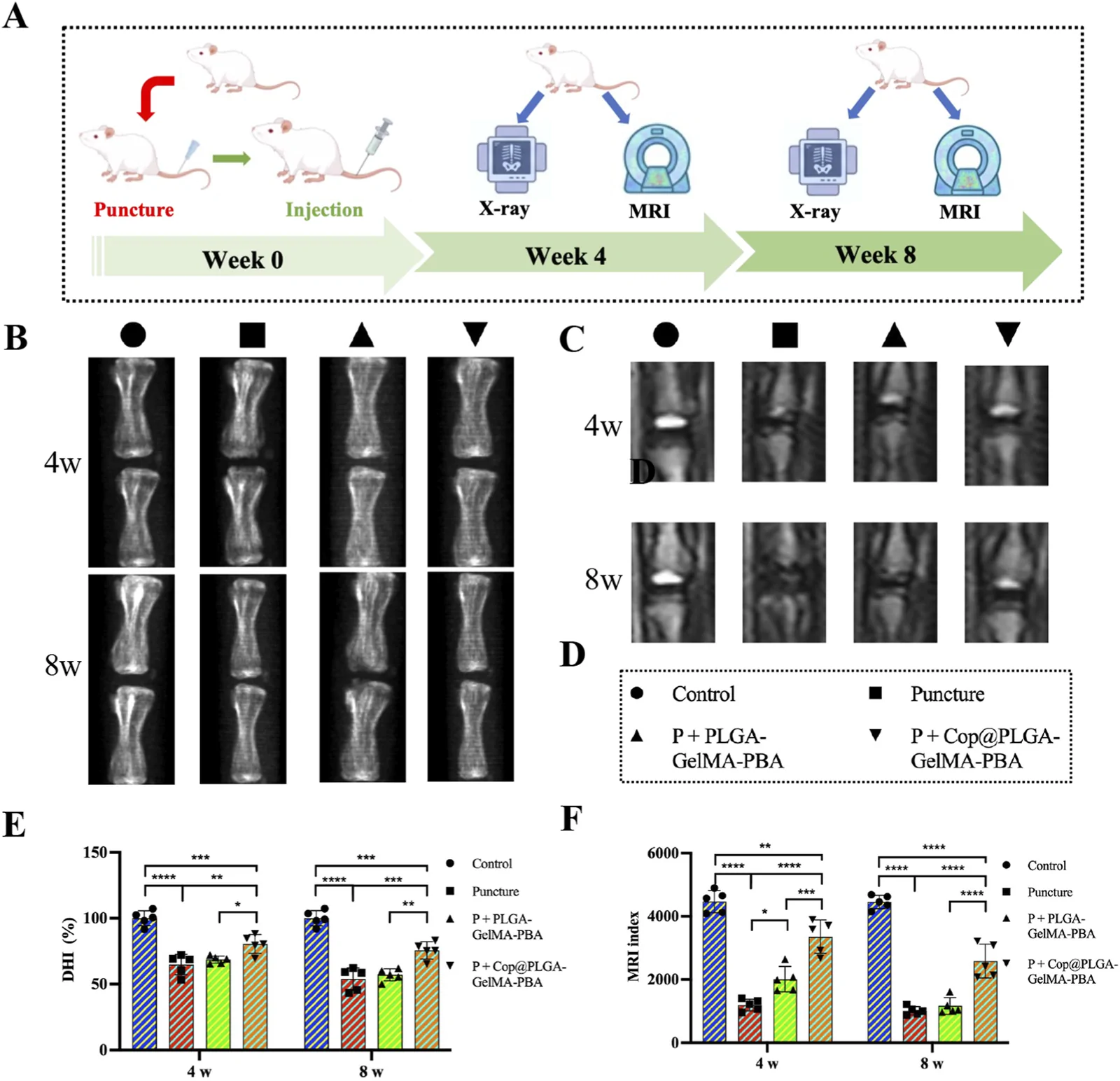
Cop@PLGA-GelMA-PBA preserves disc height and T2-weighted MRI signal after puncture. **(A)** Experimental timeline showing puncture with immediate injection and X-ray and MRI examinations at weeks 4 and 8. **(B)** Representative lateral radiographs of X-ray. **(C)** Representative T2-weighted MRI. **(D)** Group-symbol key for **(B,C)**. **(E)** Disc height index (DHI) quantification. **(F)** MRI-index quantification. All data are expressed as the mean ± SEM, *, *p* < 0.05; **, *p* < 0.01; ***, *p* < 0.001, ****, *p* < 0.0001.

### The loaded composite attenuates histological degeneration and matrix loss *in vivo*

At week 8, the Puncture group showed severe NP disruption, loss of normal architecture, and matrix depletion. Degeneration persisted in the PLGA-GelMA-PBA group, whereas the Cop@PLGA-GelMA-PBA group markedly preserved a larger NP compartment and a more organized disc structure (Figures 8A,B). Histological scores were correspondingly lower in the Cop@PLGA-GelMA-PBA group than in the Puncture group and PLGA-GelMA-PBA groups, with statistically significant differences. (Figure 8C). ACAN and Col-II fluorescence decreased markedly in the Puncture group and remained low in the PLGA-GelMA-PBA group, whereas the Cop@PLGA-GelMA-PBA group better preserved the expression of both proteins (Figures 8D–G). Thus, coptisine loading attenuated early puncture-induced degenerative changes relative to the carrier alone when delivered immediately after injury.

**FIGURE 8:**
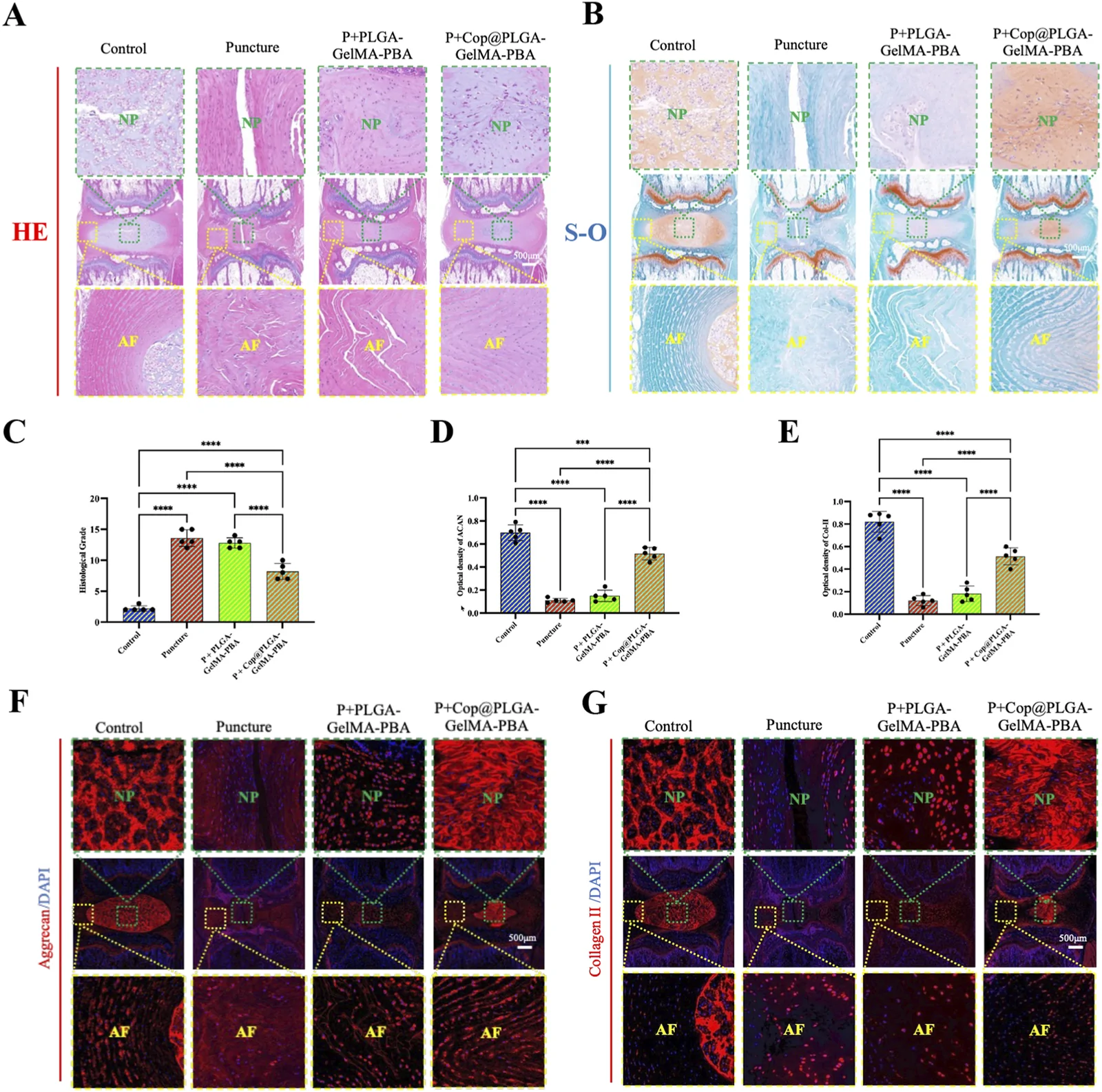
Cop@PLGA-GelMA-PBA attenuates histological degeneration and extracellular-matrix loss at week 8. **(A)** Representative H&E staining. **(B)** Representative Safranin O/Fast Green staining. **(C)** Histological degeneration scores derived from **(A,B)**. **(D,E)** ACAN and Col-II fluorescence intensity. **(F)** Representative ACAN immunofluorescence. **(G)** Representative Col-II immunofluorescence. Nuclei were counterstained with DAPI. All data are expressed as the mean ± SEM, *, *p* < 0.05; **, *p* < 0.01; ***, *p* < 0.001, ****, *p* < 0.0001.

## Discussion

In this study, we combined a key degeneration-associated molecule, a TCM-derived small molecule, and an ROS-responsive local carrier in a single discovery-to-delivery strategy. SPP1 expression increased with the severity of human NP degeneration in the present cohort, and computational screening followed by cellular testing identified coptisine as a candidate that lowered SPP1 while increasing matrix-associated genes in NPMSCs. Encapsulation of coptisine-loaded PLGA microspheres in GelMA-PBA produced a porous depot with oxidant-accelerated release and degradation. The loaded composite preserved mitochondrial and matrix-associated phenotypes during oxidative challenge and improved imaging, histological, and ECM outcomes after disc puncture. Integrating prior research (Yu et al., 2025), the contribution of this study is therefore the integration of disease-associated compound selection with NPMSC-focused validation and responsive local delivery.

SPP1 has diverse and context-dependent roles in IVD biology. During development, notochord-derived SPP1 acts as a microenvironmental cue involved in IVD formation, whereas its sustained upregulation in degenerated adult discs is associated with inflammation, extracellular matrix catabolism, and ectopic mineralization (Risbud and Shapiro, 2014). Under mechanical overload, expansion of SPP1^+^CD44^+^ notochordal cells enhances notochordal–myeloid crosstalk, promotes M1 macrophage polarization, and activates PI3K–AKT and NF-κB signaling, thereby increasing inflammatory mediators and matrix-degrading enzymes while reducing ACAN and Col-II (Risbud and Shapiro, 2014). Concurrently, SPP1 activates integrin α5β1 (ITGα5/β1) in nucleus pulposus cells and suppresses PINK1/PARKIN-dependent mitophagy, leading to damaged mitochondrial accumulation, oxidative stress, cellular senescence, and apoptosis (Molinos et al., 2015). These alterations shift nucleus pulposus cells toward catabolic and osteogenic phenotypes, ultimately promoting ECM disruption, fibrosis, and calcification (Molinos et al., 2015). Experimental inhibition of SPP1 partially restores matrix homeostasis and mitophagy while attenuating inflammation and structural degeneration in cellular and rodent models (Risbud and Shapiro, 2014; Molinos et al., 2015). Thus, persistent SPP1 activation may drive IVD degeneration through two complementary mechanisms: CD44-dependent immunoinflammatory matrix degradation and ITGα5/β1-mediated mitophagy impairment and calcification. However, its causal and therapeutic relevance in humans requires further validation (Gu et al., 2025; Zhou et al., 2023; Li Q. et al., 2026).

Importantly, these detrimental effects are not universal and appear to depend strongly on developmental stage, tissue compartment, and cellular context. During human embryonic disc formation, notochord-derived SPP1 acts as an intercellular cue within the developing NP microenvironment. Recombinant SPP1 promoted chondrogenic features and reduced VEGFA-related vascular cues in a cell-type-dependent manner, with CD44 implicated in part of this response (Zhou et al., 2023). These observations are consistent with a role for SPP1 in maintaining the cartilaginous and avascular phenotype required during disc formation. A protective role has also been reported in the adult cartilaginous endplate, where osteopontin deficiency enhanced NF-κB signaling, macrophage recruitment, and NLRP3 inflammasome activation (Wang et al., 2024). SPP1 should therefore not be regarded as intrinsically harmful throughout the disc. The direction of SPP1 activity may consequently depend on its cellular source, receptor context, extracellular concentration, disease stage, and the presence of calcification or inflammatory remodeling. Our clinical findings align with the adult degenerative NP pattern because SPP1 increased with Pfirrmann grade in the sampled NP tissues. However, we did not examine developmental tissue, endplate biology, calcification, CD44, ITGα5/β1, or mitophagic flux. The present data therefore support SPP1 as an adult NP degeneration-associated signal, not as a universal therapeutic target across every disc compartment.

Within NPMSCs, coptisine reduced SPP1 expression, whereas forced SPP1 expression weakened recovery of mitochondrial membrane potential, SOD2, Col-II, and ACAN. This reversal provides functional evidence that an SPP1-associated response contributes to the observed phenotype. It does not establish direct pharmacological binding. Natural products and TCM-derived monomers are attractive in IDD because the degenerative process is governed by linked processes rather than by a single linear pathway (Zhang K. et al., 2025). OS, mitochondrial dysfunction, inflammatory signaling, senescence, autophagy failure, ferroptosis, and ECM catabolism reinforce one another. Quercetin has reduced senescence-associated signaling through the Nrf2/NF-κB axis, while Panax notoginseng saponins have limited OS-induced NP-cell apoptosis and matrix loss (Guo et al., 2024; Shao et al., 2021). Isoginkgetin-loaded ROS-scavenging nanoparticles further illustrate how a plant-derived compound can be paired with responsive delivery to enhance autophagy and preserve NP matrix (Yu et al., 2023). Together with broader reviews (Wu et al., 2025; zhu et al., 2020), these studies support the multi-pathway potential of natural compounds. Their limitations are equally important: most evidence remains preclinical, compound composition and purity can vary, pharmacokinetics are often unfavorable, dose-response windows may be narrow, and apparent multi-target activity can complicate mechanistic attribution.

Coptisine is a quaternary protoberberine alkaloid and a characteristic constituent of Coptis chinensis (Lu et al., 2023). Its conjugated isoquinolinium structure places it within the same broad alkaloid family as berberine, while its substitution pattern gives it a distinct pharmacological profile. Studies in non-disc systems have linked coptisine to suppression of inflammatory signaling, preservation of mitochondrial function, and modulation of oxidative injury (Friedemann et al., 2015; Hu et al., 2019; Gong et al., 2012). These properties made it biologically plausible for an inflammatory and oxidative degenerative niche, but plausibility alone did not determine its selection. In the present study, coptisine emerged from a 2,987-compound library after computational prioritization and expression-based comparison of ten candidates in NPMSCs. It reduced SPP1 while increasing COL-II and ACAN, thereby connecting compound selection to the adult degenerative NP signal identified in the clinical samples.

This selection strategy also defines the evidential boundary. We used coptisine chloride at a selected working concentration and evaluated short-term NPMSC responses. We did not compare all pharmacological properties of the ten candidates, establish intracellular exposure, or define a complete dose-toxicity window. Nor do the present data show that coptisine acts exclusively through SPP1. The compound may influence several redox, mitochondrial, inflammatory, or matrix-regulatory pathways, as suggested by the transcriptomic enrichment profile and the broader pharmacology of natural products (Wu et al., 2025; Lu et al., 2023). Therefore, we consider coptisine to be an SPP1-associated, TCM-derived candidate rather than a selective SPP1 inhibitor. Local formulation is intended to improve payload retention within the transport-limited disc (Wang et al., 2026; Gorth et al., 2012), but it does not eliminate the need for pharmacokinetic, toxicological, and target-engagement studies.

The choice of carrier was guided by the physical and biochemical constraints of the degenerating disc. The NP is avascular, densely hydrated, and enclosed by the annulus fibrosus and cartilaginous endplates, which restrict transport (Urban et al., 2004). PLGA microspheres can protect a payload and prolong release, whereas GelMA-based networks can provide a photocrosslinkable local depot (Xiong et al., 2025; Nie et al., 2025). Embedding Cop@PLGA within GelMA-PBA was therefore intended to combine microsphere-level sustained release with hydrogel-level localization and microenvironment responsiveness. OS provides the pathological trigger for this design. Degenerative NP cells experience mitochondrial dysfunction and excessive ROS (Wang Y. et al., 2023), which can amplify NF-κB and inflammasome signaling, senescence, ferroptosis, and matrix catabolism (Guo et al., 2025; Chen et al., 2022; Gu et al., 2025). Phenylboronic acid groups can undergo ROS-dependent oxidation, destabilizing responsive linkages (Zeng et al., 2021; Kim et al., 2017). In principle, this allows faster matrix erosion and payload release where OS is greatest. Related systems have used pathological-microenvironment-responsive GelMA-based hydrogels, ROS-cleavable PBA hydrogels, ROS-responsive magnesium microspheres, and thermosensitive ROS-responsive depots to regulate inflammation, ferroptosis, oxidative injury, or secretory autophagy in experimental IDD (Gao et al., 2024; Liu et al., 2024; Zhang et al., 2023; Zheng et al., 2021). These studies support ROS as a rational release cue rather than merely a target to be suppressed.

Our material data are consistent with this concept: TBHP accelerated both coptisine release and composite mass loss, in keeping with established oxidation-sensitive PBA chemistry (Zeng et al., 2021; Kim et al., 2017). Microsphere incorporation preserved a porous architecture and short-term cytocompatibility. The unloaded scaffold produced substantially less benefit than the loaded composite *in vivo*, supporting a contribution from coptisine. TBHP was selected as a stable, water-soluble organic hydroperoxide that provides reproducible *in vitro* oxidation conditions and serves as a practical ROS surrogate. However, endogenous disc ROS are chemically heterogeneous; in addition to peroxides, they include superoxide (O_2_•−) and hydroxyl radical (•OH). Nevertheless, TBHP is an *in vitro* oxidant surrogate and does not reproduce the concentration, spatial distribution, or temporal fluctuations of ROS in a degenerating disc. Accordingly, the present findings demonstrate TBHP-responsive behavior under the tested conditions, whereas the responsiveness of Cop@PLGA-GelMA-PBA to other ROS species and their concentrations *in vivo* remains to be determined. During H_2_O_2_ challenge, Cop@PLGA-GelMA-PBA partially restored mitochondrial polarization, SOD2 abundance, and matrix proteins. The attenuation of these responses by SPP1 overexpression supports functional involvement of SPP1-associated signaling. This observation is biologically compatible with reports linking elevated SPP1 to impaired mitophagy and ROS accumulation in degenerative NP cells (Gu et al., 2025).

Transcriptomic profiling placed the cellular response in a broader biological context. The enrichment of cytokine–cytokine receptor interaction, TNF, chemokine, MAPK, IL-17, NF-κB, AGE–RAGE, integrin, cell-adhesion, and ferroptosis-related pathways is consistent with the involvement of inflammatory, oxidative, and extracellular-matrix-remodeling programs in IDD (Risbud and Shapiro, 2014; Molinos et al., 2015; Chen et al., 2022). However, over-representation analysis does not determine whether these pathways are activated or suppressed, nor does it establish a causal anti-inflammatory effect of Cop@PLGA-GelMA-PBA. Because inflammatory cytokines, pathway-specific proteins, and functional inflammatory readouts were not assessed in the present study, these findings should be interpreted as hypothesis-generating and require targeted validation.

The focus on NPMSCs distinguishes the present biological question from studies of mature NP cells. Resident progenitor and mesenchymal populations decline or become dysfunctional during degeneration (Sakai et al., 2012; Li Z. et al., 2026; Zhang Y. et al., 2025). The isolated rat cells exhibited trilineage differentiation and a qualitative CD34-low/CD45-low/CD73-high/CD90-high/CD105-high profile. Coptisine increased matrix-associated transcripts, and the loaded material preserved matrix proteins during oxidative challenge. The approach therefore seeks to protect endogenous repair-associated cells rather than replace them or directly extrapolate mechanisms established in mature NP cells. The animal findings were consistent across imaging, histology, and matrix markers. Puncture caused progressive loss of disc height and T2-weighted signal, followed by severe architectural and ECM deterioration. The PLGA-GelMA-PBA produced limited improvement, whereas Cop@PLGA-GelMA-PBA preserved DHI, MRI index, disc organization, ACAN, and Col-II. Because the material was injected immediately after puncture, these data support early attenuation of injury-induced degeneration within established puncture-model frameworks (Han et al., 2008), not repair of an established degenerative disc (Gullbrand et al., 2018).

Notably, rather than delaying treatment until 2 weeks after puncture as in previous studies, we administered Cop@PLGA-GelMA-PBA immediately after injury. This early intervention allowed us to assess whether coptisine enhances the antioxidant capacity of NPMSCs before the IVD tissue becomes relatively stabilized (Masuda et al., 2005). Moreover, because the extent of IDD is not necessarily proportional to pain severity and most patients with low back pain do not present with advanced disc degeneration (Kim et al., 2011), treatment at an early stage of degeneration may better resemble the clinical setting. Thus, immediate administration may offer greater clinical relevance by preserving discs before substantial degeneration occurs.

This study has several limitations. First, our drug discovery pipeline relied on *in silico* virtual screening rather than experimental high-throughput screening with large-scale physical libraries. Consequently, the subsequent validation involving only a small cohort of compounds may have led to the omission of potentially efficacious candidates. Second, while bioinformatics analysis implicated SPP1, direct coptisine–SPP1 binding remains to be experimentally confirmed via biophysical assays. Third, the animal study used an acute puncture-induced rat model with immediate post-injury injection, which evaluates early intervention rather than therapeutic efficacy in established IDD and does not fully recapitulate the chronic, multifactorial pathophysiology of human disease; therefore, validation in delayed-treatment and large-animal models is warranted.

## Conclusion

In this work, the TCM-derived small molecule coptisine, delivered from an ROS-responsive PLGA-GelMA-PBA material, preserved NPMSC mitochondrial and matrix-associated phenotypes and attenuated early injury-induced disc degeneration after puncture in rats. SPP1 served as a context-dependent, adult degeneration-associated signal for compound selection and contributed functionally to the cellular response. The study provides a preclinical basis for integrating disease-associated small-molecule selection with microenvironment-responsive local delivery.

## Data Availability

The raw data supporting the conclusions of this article will be made available by the authors, without undue reservation.
